# Are APACHE II scores better predictors of mortality than routine laboratory values?

**DOI:** 10.1186/cc12411

**Published:** 2013-03-19

**Authors:** Z Baykara, H Özocak, A Kuş, Z Arslan, B Yüksel, C Aksu, M Ertagin, M Solak, K Toker

**Affiliations:** 1 University of Kocaeli, Turkey

## Introduction

Even though more accurate and updated versions such as APACHE III and IV have been developed, APACHE II has remained the most widely used severity scoring system in the ICU [1]. In the present study, we aimed to develop a model mainly based on the laboratory data available upon admission to predict mortality and to compare its performance with that of APACHE II in a mixed ICU in Turkey.

## Methods

A total of 645 adult patients who were over 18 years old and who stayed in the ICU for more than 24 hours were included in this study. Using stepwise multivariate logistic regression analysis, a mortality prediction model was developed based on the laboratory data, diagnostic category and age. The adjusted probability of death, according to the diagnostic category of the APACHE II score (adj-APACHE II), was calculated. The ability of the laboratory model and the adj-APACHE II model to discriminate between survivors and nonsurvivors was assessed using receiver operating characteristic (ROC) curves. The calibration of the laboratory model and the adj-APACHE II model was assessed by the Hosmer and Lemeshow goodness-of-fit test.

## Results

The areas under the ROC curves of the laboratory models and the adj-APACHE II scores for the prediction of mortality were 0.80 (95% CI: 0.76 to 0.85) and 0.78 (95% CI: 0.74 to 0.83), respectively (*P >*0.05, *z *statistic). The Hosmer-Lemeshow statistic had a chi-squared value of 2.01 (*P *= 0.98) for the laboratory model and 13.2 (*P *= 0.10) for the adj-APACHE II model.

## Conclusion

If the aim is to predict mortality as accurately as APACHE II, the mortality prediction model based mainly on routine admission laboratory tests can achieve this using computer technology, without labor-related costs, as soon as the patient is admitted to the ICU. However, it is necessary to perform multicenter validation studies.
